# *Salmonella* spp. Response to Lytic Bacteriophage and Lactic Acid on Marinated and Tenderized Raw Pork Loins

**DOI:** 10.3390/foods11060879

**Published:** 2022-03-19

**Authors:** Sherita Li, Haley M. Konoval, Samantha Marecek, Amanda A. Lathrop, Siroj Pokharel

**Affiliations:** 1 Food Science & Nutrition Department, California Polytechnic State University, San Luis Obispo, CA 93407, USA; sli45@calpoly.edu (S.L.); lathrop@calpoly.edu (A.A.L.); 2 Animal Science Department, California Polytechnic State University, San Luis Obispo, CA 93407, USA; hkonoval@calpoly.edu (H.M.K.); smarecek@calpoly.edu (S.M.)

**Keywords:** bacteriophage, lactic acid, *Salmonella* spp., pork loins, food safety

## Abstract

Bacterial food poisoning cases due to *Salmonella* have been linked with a variety of pork products. This study evaluated the effects of a *Salmonella*-specific lytic bacteriophage and lactic acid (LA) on *Salmonella* Enteritidis, *Salmonella* Montevideo, and *Salmonella* Heidelberg growth on raw pork loins. Pork loins were cut into approximately 4 cm thick slices. Pork slices were randomly assigned to five treatment groups (control, DI water, LA 2.5%, phage 5%, and LA 2.5% + phage 5%) with six slices per group per replication. Pork loins were inoculated with 10^6^ CFU/mL of *Salmonella* spp. and stored at 4 °C for 30 min. After 1 h of treatment application and marination, phage 5% significantly (*p* < 0.05) reduced the surface bacterial population by 2.30 logs when compared with the control group. Moreover, the combined treatment of LA 2.5% + phage 5% significantly (*p* < 0.05) reduced the surface bacterial population by more than 2.36 logs after 1 h of marination. In the post-tenderization surface samples, the combination of both phage and LA showed a significant reduction (*p* < 0.05) when compared with the control group. However, the treatments had no effect (*p* > 0.05) when analyzing the translocation of pathogens on pork loins.

## 1. Introduction

Pork is the most widely eaten meat globally, and recent data show that consumption has more than doubled in developing countries [1,2]. Furthermore, the Food and Agricultural Organization of the United Nations (FAO) Food Balance Sheet data show that U.S. pork consumption will increase over time [3]. Because pork consumption will continue to grow, ensuring microbial safety should be a top priority.

*Salmonella* continues to be a leading cause of domestically acquired foodborne illness resulting in hospitalization, which indicates that more research needs to be conducted [4]. Likewise, there are 31 pathogens known to cause food poisoning, and *Salmonella* spp. continues to be one of the most common bacterial food contaminants over time [5]. Among the 2300 serotypes, *Salmonella enterica* serovar Enteritidis accounts for almost half of all human *Salmonella* infections worldwide [6]. In 2019, the Centers for Disease Control and Prevention (CDC) investigated 13 multistate *Salmonella*-related outbreaks that infected over 1100 people from 49 states and caused two deaths. [7]. While the CDC data show total illnesses and not specific cases attributable to meat consumption, *Salmonella* is often linked to outbreaks detected in meat and meat products [8]. Likewise, in the European Union, food associated with the consumption of pork and meat products causes 9.8% of *Salmonella* and 4% of *Salmonella* Enteritidis outbreaks [9]. As a result, research related to the improvement of food safety is needed and can provide long-term economic advantages to the meat industry [10].

*Salmonella* infection causes salmonellosis and manifests into several disease syndromes, including gastroenteritis, septicemia, and typhoid fever [11]. Most cases of salmonellosis are mild and self-limiting in healthy people. However, sometimes it can be life-threatening, particularly in young children, the elderly, and immunocompromised people [12]. The development of gastroenteritis often starts 6–72 h following the ingestion of contaminated water or food [13]. Most people with severe illnesses from salmonellosis are commonly treated with antibiotics [14]. However, the emergence of multi-drug-resistant (MDR) *Salmonella* serotypes is a global public health concern and poses a severe threat to humans through the food industry [15].

*Salmonella* has evolved to develop new genes to help it to withstand challenging environments, settle into a new environmental niche, and establish antimicrobial resistance [16]. In a recent outbreak of 37 cases, a cluster of multi-drug-resistant monophasic *S*. Typhimurium was identified as being likely associated with pork consumption [17]. The development of antimicrobial resistance genes is an increasing threat to public health and has led to research in alternative practices to eliminate pathogenic bacteria. In recent years, food safety scholars have been looking at the effect of lytic bacteriophages and their likelihood of improving food safety. Bacteriophages are natural predators of bacteria and can replicate and harm the targeted bacterium.

Meanwhile, the United States Food and Drug Administration (US-FDA) has approved several phage preparations for meat and poultry products as a food processing aid [18]. Numerous successful studies have shown that phages can indicate significant antimicrobial resistance, but their transduction abilities can be tested prior to application [19,20]. Likewise, a natural antimicrobial lactic acid (LA) has been widely used in the meat and poultry industries against foodborne pathogens. The introduction of lytic bacteriophages and LA as antimicrobial treatments in pork containing the harmful *Salmonella* spp. could help to increase the food safety of meat products.

Lytic bacteriophages and lactic acid on poultry products have proven effective in multiple studies [21,22]. However, there is not as much research on pork, which is known to contain the pathogen *Salmonella* and other *Salmonella enterica* subspecies that can cause illnesses or hospitalization [23]. Therefore, testing lytic phages and lactic acid in pork can help gather more data on the efficacy of these treatments. This study aims to determine the efficacy of lytic bacteriophages and LA to control the growth of *Salmonella* Enteritidis, *Salmonella* Montevideo, and *Salmonella* Heidelberg in marinated and tenderized raw pork loins.

## 2. Materials and Methods

### 2.1. Raw Pork Loins

Raw pork loins were procured from a nearby grocery store and were transported under ice (<4 °C) to the food safety lab at Warren J. Baker Center for Science and Mathematics, Cal Poly. Upon arrival, the loins (pH 5.6–5.8) were portioned into ~4 cm thick slices ranging from 200 to 300 g per piece (5 cm × 10 cm; *n* = 6 per treatment; 30 total per replication) and randomly allocated to different treatment groups. Then, randomly assigned slices were prepared for inoculation and further processing.

### 2.2. Meat Marination

A standard marination solution was prepared to deliver 0.35% sodium chloride (Morton Salt, Chicago, IL, USA) and 0.45% sodium tripolyphosphate (85% STPP–Na_5_O_10_P_3_, Acros Organics, Janssen Pharmaceuticalaan, Geel, Belgium) in a final product at a targeted 10% uptake level. Based on the meat weight, a batch of the marinade was made on the same day and equally divided for five different treatments. Marinade pH was measured prior to (7.2) and after one hour of marination (6.8).

### 2.3. Bacterial Strain and Culture Preparation

Three *Salmonella* strains associated with previous outbreaks were used in this study (Table 1). Pure cultures from previously isolated specimens of *Salmonella* Enteritidis (*S*. Enteritidis), *Salmonella* Montevideo (*S*. Montevideo), and *Salmonella* Heidelberg (*S.* Heidelberg) were obtained from the Department of Food Science and Nutrition, California Polytechnic State University (Cal Poly), San Luis Obispo, California. Throughout the experiment, strains were individually maintained on Tryptic Soy Broth (BD Bacto™ Tryptic Soy Broth, Fisher Scientific, Pittsburgh, PA, USA).

To prepare a cocktail for meat inoculation, individual strains were grown separately in 10 mL of tryptic soy broth (TSB) and passaged three times at 37 °C for 18 to 24 h to reach a final concentration of 1.0 × 10^8^ CFU/mL. Later, the cocktail was prepared in buffered peptone water (BPW-Oxoid Ltd., Basingstoke, Hants, UK) by mixing the three strains. Throughout the experiment, a final concentration of 1.0 × 10^6^ CFU/mL of *Salmonella* cocktail was maintained in the BPW broth.

### 2.4. Inoculation of Loins

**Figure 1:**
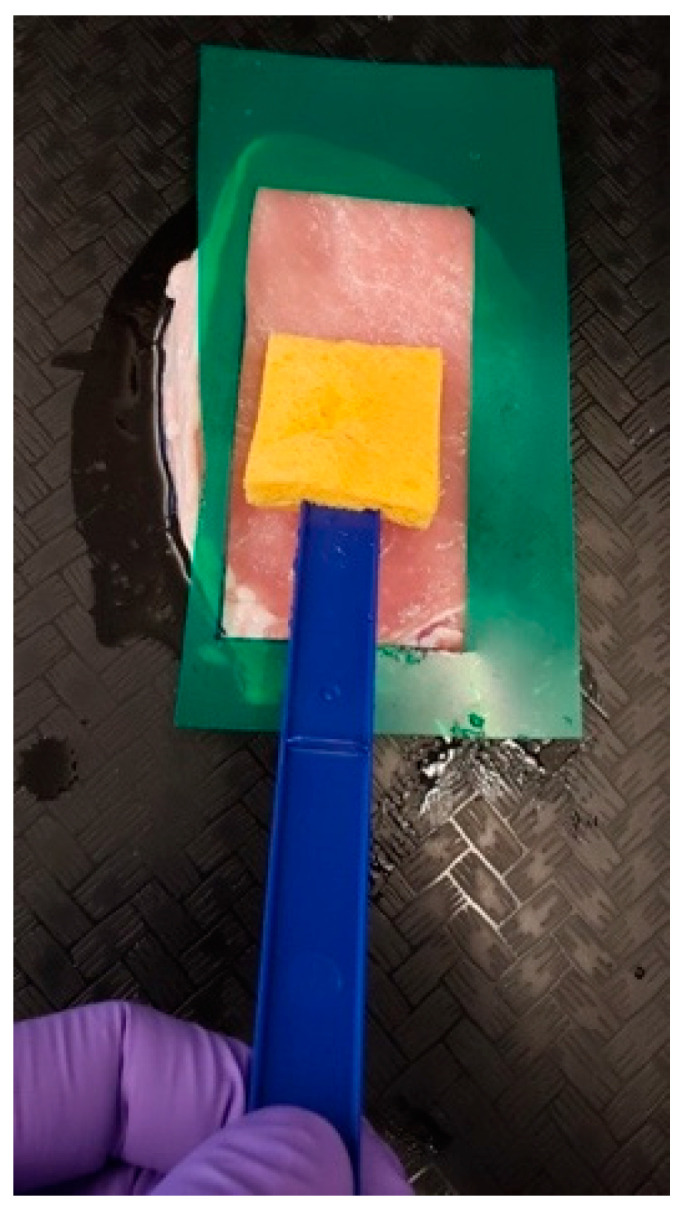
Meat surface swabbing using a sponge moistened in BPW (buffered peptone water).

Pork slices from loins (pork samples) were experimentally inoculated in the cocktail (10^6^ CFU/mL) of *S.* Enteritidis, *S.* Montevideo, and *S.* Heidelberg for two minutes following the dip inoculation method. After inoculation, slices were left for 30 min attachment at 4 °C. Later, pork slices were placed in a plastic container containing 0.35% sodium chloride and 0.45% sodium triphosphate to be marinated for 1 h. Plastic templates (5 cm × 10 cm) were used, along with microbial sponge sticks (EZ Reach™ Sponge Sampler, World Bioproducts, Libertyville, IL, USA), to swab the pork slice surface (Figure 1).

### 2.5. Treatment Plan

Raw pork slices were randomly assigned to one of the five treatments (Control, DI (Deionized) water, 2.5% LA (lactic acid), 5% phage, 2.5% LA + 5% phage; *n* = 6) per replication. Before the treatment application, pork slices were first kept in the marinade. The solutions of 5% (1 × 10^8^ PFU (plaque-forming unit)/mL on meat surface) lytic bacteriophages (PhageGuard^®^ S, Micreos Food Safety, The Hague, The Netherlands) and 2.5% LA (Fisher Scientific, Pittsburgh, PA, USA) were prepared based upon the manufacturer’s recommendation of 10 μL/cm^2^ surface application. Lactic acid and bacteriophage applications were performed by pipetting the required volume of each treatment onto the pork slices. Treated pork slices were kept at refrigeration temperature with marinade for 1 h before sampling.

### 2.6. Swab Collection

After the application of treatments, slices were stored at 4 °C for 1 h prior to sampling. Microbial sponge sticks were used for a swab collection from each raw pork portion (Figure 1). One pork slice from each treatment group was randomly selected to be removed after 30 min and processed for initial surface attachment of *Salmonella* spp. Three loin slices were then randomly selected within each treatment group after 1 h to be removed and sampled for post-treatment surface attachment. Before swabbing the meat surface for *Salmonella*, the sponge was squeezed inside the bag to remove the excess BPW; the swab was then taken from inside a template (50 cm^2^) of the raw pork slice. The sponge stick was returned to the collection bag and placed in the Stomacher^®^ (Model 400 circulator, Seward, London, UK) at 230 rpm for two minutes. Serial dilutions were then plated onto XLD (Xylose Lysine Deoxycholate agar: Oxoid Ltd., Basingstoke, Hants, UK) agar plates in duplicate, incubated at 37 °C, and typical *Salmonella* colonies were enumerated (CFU/cm^2^).

### 2.7. Translocation Study

**Figure 2:**
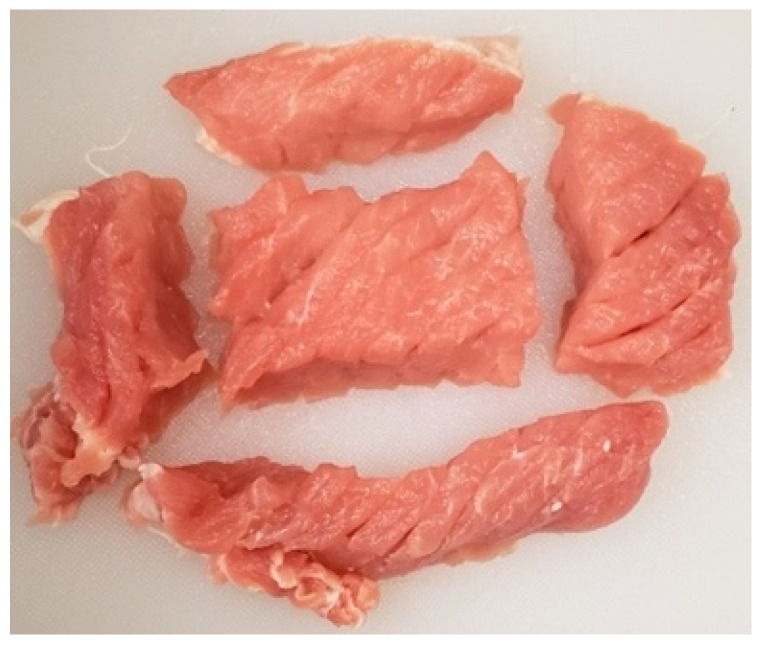
Meat internal core prior to the flaming procedure.

Two remaining pork loin slices were tenderized after 1 h of refrigeration using a manual meat tenderizer (model 7-3101-w, Weston, Zhejiang, China) for the bacterial translocation study. For the entirety of the experiment, one manual tenderizer was used and sterilized between treatments. Tenderized samples were then swabbed over the entire surface using a sponge stick moistened with 10 mL BPW. A template (50 cm^2^) was used to swab the entire surface, and the sponge was placed in the stomacher at 230 rpm for 2 min. After swabbing the surface, a sterile subsection from the centermost portion of the slice was obtained using flame-sterilized knives (Figure 2). The sterilization and flaming procedure followed the methods described by Muras et al., 2012 [24]. Sterilized cores were placed into a food processor (Cuisinart, Stamford, CT, USA). Two 25 g aliquots from processed samples within each treatment group were collected and individually placed in a sterile filtered bag. Samples were paddle-blended with 225 mL tryptic soy broth (TSB) using a Stomacher^®^ at 230 rpm for 2 min.

Dilutions were plated in duplicate on XLD agar for each of the treatments and incubated at 35 ± 2 °C for 24 h. Presumptive positive colonies from each plate were sub-cultured on lysine iron agar (Thermo Scientific™, Oxoid Deutschland GmbH, Wesel, Germany) and triple sugar iron (TSI, Thermo Scientific™, Remel, San Diego, CA, USA) for purification and biochemical confirmation. Before the statistical analysis, *Salmonella* plate counts were log_10_ transformed and reported.

### 2.8. Research Design and Statistical Analysis

The experiment was replicated three times and designed as a randomized complete block design. The repeated replication served as different blocks. Treatments were assigned at random within the blocks. Storage length and marination length served as independent variables. Response variables of interest included bacterial surface attachment (30 min surface swabs, 1 h surface swabs, post-tenderization surface swabs), meat pH, and internal pathogen concentration (1 h post-tenderization cores). Before statistical analysis, bacterial cell counts were converted to log_10_ CFU/cm^2^ for surface and log_10_ CFU/g for cores. Data were analyzed using the PROC MIXED procedure of SAS (v. 9.4, SAS Inst., Cary, NC, USA). The least-square means were generated using the LSMEANS statement, separated using the PDIFF function and considered significant at an α of 0.05.

## 3. Results

The initial surface attachment of *Salmonella* spp. on randomly assigned loin slices to the different treatments prior to application ranged from 3.30 to 4.55 log_10_ CFU/cm^2^ (Table 2). The loin slices assigned to the lactic acid treatment were significantly different at the initial attachment, which could be due to the difference in the surface area. Lytic bacteriophage (5%) was able to significantly reduce (*p* < 0.05) the surface microbial population in the marinated pork loins by 2.3 logs in one hour compared with the control group. However, it should be noted that the control group also experienced a decrease in CFU from the initial surface attachment of 4.55 logs to 3.90 logs after 1 h. In addition, the combination of both lactic acid (2.5%) and lytic bacteriophage (5%) on the pork loins also resulted in a significant reduction (*p* < 0.05) in *Salmonella* spp. from the surface by 2.35 logs after 1 h of marination (Table 2).

Likewise, a significant reduction (*p* < 0.05) in *Salmonella* spp. on surface attachment post-tenderization was observed when 5% phage and the combination of 2.5% LA + 5% phage was used on the pork loins (Table 2). Out of all five treatments, the combination of 2.5% lactic acid and 5% lytic bacteriophage showed the most reduction in marinated pork loins. However, the rest of the treatments do not significantly differ (*p* > 0.05) in the *Salmonella* spp. population on the post-tenderized loin surface (Table 2). Phage application combined with lactic acid alone showed that the lytic bacteriophage could reduce the surface-attached foodborne pathogens.

**Figure 3:**
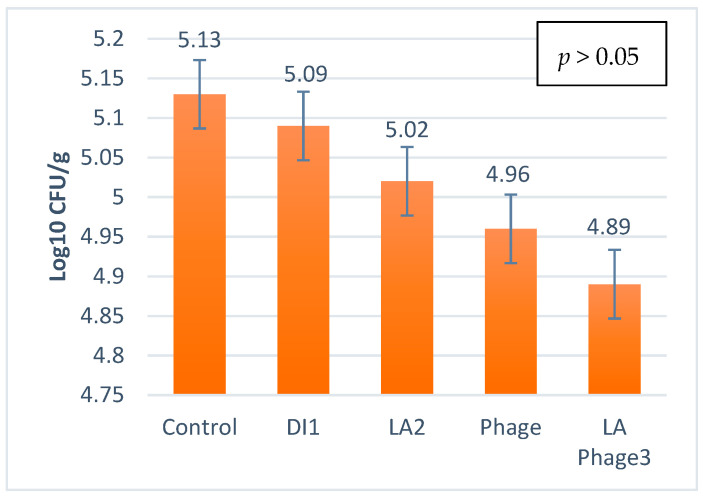
The influence of lactic acid (2.5%), lytic bacteriophage (5%) and a combination of both in marinated and manually tenderized pork slice cores inoculated with *Salmonella* spp. DI1: Deionized water; LA2: Lactic acid; LA Phage3: Lactic acid phage

Similarly, when comparing different interventions, the 5% phage along with the 2.5% LA + 5% phage showed significant (*p* < 0.05) reductions in surface-attached *Salmonella* spp. after 1 h of pork marination compared with the control. After 1 h, the 5% phage and combination of 2.5% LA + 5% phage were able to reduce the surface-attached pathogen by 1.72 and 1.77 logs, respectively (Table 2). However, for the post-tenderization surface attachment, none of the interventions significantly reduced the surface attachment of *Salmonella* spp., except for the LA + phage treatment, which showed a lower log reduction (Table 2) when compared with the control. For the bacterial translocation study, the control group showed an increase in the amount of *Salmonella* spp. translocated to the internal cores from the surface after manual tenderization. However, there was no significant treatment effect (*p* > 0.05) on the translocation of *Salmonella* spp. measured in log_10_ CFU/g in the internal cores (Figure 3). After manual tenderization of pork loins, a high number of *Salmonella* spp. was isolated from the internal cores.

## 4. Discussion

The present study was conducted to provide more information on the efficacy of lytic bacteriophages and lactic acid in an experimental *Salmonella* inoculation in raw pork loins. Because of the growing antibiotic resistance among different *Salmonella* serovars, the experiments on non-antibiotic treatments, such as lytic bacteriophages and lactic acid for reducing *Salmonella* concentrations [25,26], have garnered some interest.

Lactic acid is a natural acid that continues to be widely used in the food industry as an antimicrobial treatment against foodborne pathogens [27]. Research determined that the presence of *Salmonella* spp. was lower on pork carcasses sprayed with a 2% lactic acid solution than those that were not sprayed, but an actual reduction was not reported [28]. However, it was observed that *Salmonella* was able to adjust to acidic environments and survive in unfavorable pH conditions by developing a significant acid-tolerance behavior through increasing the expression of genes, including *rpoS, nlpD* and *clpP* [29]. According to a study performed by Alvarez- Ordóñez, et al. [30], *S.* Typhimurium was still able to grow within the temperature range of 25–37 °C when the pH was reduced to 5.4 using lactic acid. Therefore, the acid tolerance in *Salmonella* could decrease the efficacy of the LA treatment in this study.

Due to the unique ability to infect and lyse specific bacterial cells, bacteriophage applications have become a suitable food safety invention for the food industry [25]. Many studies demonstrated the efficacy of bacteriophages on *Salmonella* in different food matrixes. Wang et al. (2017) [31] reported a 2.3 log CFU/g reduction in *Salmonella* counts in fresh, chilled pork. A consistent 1 log reduction in *Salmonella* load has been reported in four meat types (beef, pork, chicken, and turkey) when applying bacteriophages on trim and thighs prior to grinding [32], which is consistent with our study.

In this study, the antimicrobial treatments did not significantly reduce *Salmonella* spp. when studying the translocation of the pathogen post-tenderization. Possibly, higher dosages of lytic bacteriophage and lactic acid would substantially decrease microbial translocation on pork loins. More research is needed to evaluate the correct dosage of lytic bacteriophage combined with lactic acid to considerably reduce the translocated *Salmonella* spp. in pork meat. In theory, manual tenderization with the help of a tabletop tenderizer and increased marination length would enhance pathogen internalization on meat products. Previous studies have shown that microbial internalization is possible on various processing technologies [33,34,35]. Likewise, other studies have shown that moisture content significantly influences the survivability of *Salmonella* [36,37]. Perhaps not covered by the current research, Calle et al. (2015) [38] demonstrated that *Salmonella* populations increased 2.0 log CFU/g in tenderized rib eyes cooked to 37.8 °C when held at 60.0 °C for 8 h. Therefore, mechanical tenderization, shelf life, and temperature are all factors that may pose a food safety risk to consumers in terms of inadequate lethality and/or subsequent outgrowth of *Salmonella*.

## 5. Conclusions

Lytic bacteriophages and lactic acid as antimicrobial interventions effectively reduce *Salmonella* on the surface of pork loin. This research has found that the combination of 5% lytic bacteriophage and 2.5% lactic acid significantly reduced the *Salmonella* cocktail (*p <* 0.05) on raw pork loin meat surfaces by 1.75 logs but failed to significantly reduce *Salmonella* spp. in the internal cores of pork loin after manual tenderization. According to the data, lactic acid is not a suitable treatment for pork loins but could be an option if combined with lytic bacteriophages. While not formally assessed, minimal negative impacts were caused by the usage of lactic acid and lytic bacteriophages on pork loins regarding product color. The results from this study have a significant influence on the reduction in *Salmonella* prevalence in pork and can be applied to the U.S. pork industry, extending to domestic and export markets. The reduction in *Salmonella* spp. on raw pork loins will enhance food safety and reduce the number of foodborne infections.

## Figures and Tables

**Table 1:** *Salmonella* strains used in this research and their previous outbreak sources.

Bacterial Strain Name	Strain Number	Isolate
*Salmonella* Enteritidis	FSL S5–415–ILSI NA	Human isolate
*Salmonella* Heidelberg	FSL S5–448–ILSI NA	Human isolate
*Salmonella* Montevideo	FSL S5–630–ILSI NA	Bovine isolate

FSL: Food Safety Lab at California Polytechnic State University, San Luis Obispo, CA, USA; ILSI NA: International Life Science Institute North America culture collection, Cornell, Ithaca, NY, USA.

**Table 2:** The influence of lactic acid (2.5%), lytic bacteriophage (5%), and a combination of both on the surfaces of marinated and manually tenderized pork loins inoculated with *Salmonella* spp.

Bacterial Attachment (Log_10_ CFU cm^−2^)	Interventions					*SEM* ^4^
	Control	DI ^1^	LA ^2^	Phage	LA Phage ^3^	
Initial surface attachment (30 min)	4.55 ^Aa^	4.31 ^Aa^	3.30 ^Bb^	4.48 ^Aa^	4.48 ^Aa^	0.20
Surface attachment (1 h)	3.90 ^Ab^	3.97 ^Aab^	3.86 ^Aa^	2.18 ^Bc^	2.13 ^Bc^	0.11
Surface attachment post-tenderization	3.67 ^Ab^	3.57 ^Ab^	3.53 ^Aab^	3.22 ^ABb^	3.03 ^Bb^	0.17

^A,B^ Means lacking common superscript letters in the same row are different (*p* < 0.05). ^a,b,c^ Means lacking common superscript letters in the same column are different (*p* < 0.05). ^1^ DI: deionized water; ^2^ LA: lactic acid; ^3^ LA Phage: lactic acid phage; ^4^ *SEM*: standard error of the mean.

## Data Availability

Not applicable.
